# Current approach in the diagnosis and management of posterior uveitis

**DOI:** 10.4103/0301-4738.58470

**Published:** 2010

**Authors:** S Sudharshan, Sudha K Ganesh, Jyotrimay Biswas

**Affiliations:** 1 Medical Research Foundation, 18, College Road, Sankara Nethralaya, Chennai – 600 006, India; 2 Medical and Vision Research Foundation, 18, College Road, Sankara Nethralaya, Chennai – 600 006, India

**Keywords:** Goldman–Witmer co-efficient, immunosuppressives, infective posterior uveitis, ocular toxoplasmosis, ocular toxocariasis, ocular tuberculosis, polymerase chain reaction, systemic steroids, white dot syndromes

## Abstract

Posterior uveitic entities are varied entities that are infective or non-infective in etiology. They can affect the adjacent structures such as the retina, vitreous, optic nerve head and retinal blood vessels. Thorough clinical evaluation gives a clue to the diagnosis while ancillary investigations and laboratory tests assist in confirming the diagnosis. Newer evolving techniques in the investigations and management have increased the diagnostic yield. In case of diagnostic dilemma, intraocular fluid evaluation for polymerase chain testing for the genome and antibody testing against the causative agent provide greater diagnostic ability.

Posterior uveitis can commonly be insidious in onset although it can have an acute presentation. This sight-threatening condition has pathognomonic clinical features identifiable on clinical examination. According to the Standardisation of Uveitis Nomenclature working group[1] classification, posterior uveitis is classified as given in Table 1.

**Table 1:** SUN working group classification of uveitis and the primary site of inflammation

Type	Primary site of inflammation	Includes
Anterior uveitis	Anterior chamber	Iritis, iridocyclitis, anterior cyclitis
Intermediate uveitis	Vitreous	Pars planitis, posterior cyclitis, hyalitis
Posterior uveitis	Retina/choroid	Focal, multifocal, diffuse choroiditis, chorioretinitis, retinochoroiditis, retinitis, neuroretinitis
Panuveitis	Anterior chamber, vitreous and retina or choroid	

Posterior uveitis can have inflammation involving adjacent structures such as the retina, vitreous, optic nerve head, retinal vessels, along with choroidal inflammation.

A thorough diagnostic work-up directed by the history of presenting complaints, patient's symptoms and signs, and clinical examination is mandatory. Ancillary investigations such as fundus fluorescein angiography (FFA), indocyanine green angiography (ICG), ultrasonography (USG), optical coherence tomography (OCT), and selective laboratory investigations help in confirming the diagnosis. It is of paramount importance to identify the possible etiology as posterior uveitis can be infective or non-infective. Newer diagnostic modalities have led to early recognition of the condition and its possible etiology, which has a positive influence on the management of the disease. This article gives an overview of the current approach in the diagnosis and management of common posterior uveitic entities found in India.

## Clinical approach to a patient of posterior uveitis

**History:** Thorough history is a critically important step in evaluating any patient with uveitis. It allows the clinician to gain critical evidence regarding general medical history, travel history, social history, associated medications and family history. These often provide critically useful information.

**Critical questions for diagnosis:** Answers to the following set of questions paves the way for an accurate diagnosis and management of posterior uveitic entities. It is also helpful in identifying the possible etiology enabling appropriate treatment.

1. Is it posterior uveitis only or is it part of a panuveitis?
2. Is it choroiditis, retinitis, or retinochoroiditis?
3. Is there associated involvement of the optic nerve head and/or the retinal vessels?
4. Does the clinical feature fit into any known infective or non-infective entity?
5. Is there associated anterior segment inflammation, vitritis, or complications?
6. Is it associated with other systemic features?
7. Is it recurrent? If so, how has it responded to previous therapy?
8. Is it associated with an immunocompromised state?
9. Is it a masquerade syndrome?

### Posterior uveitic diseases can be classified based on

#### 1. Etiology

1. Infective causes
  Toxoplasmosis
  Toxocariasis
  Tuberculosis (TB)
  Syphilis
  Bartonella
  Viral (*Herpes simplex*, *Varicella zoster*, cytomegalovirus [CMV])
  Human immunodeficiency virus (HIV)-related eye diseases
2. Non-infective causes
  Acute posterior multifocal placoid pigment epitheliopathy (APMPPE)
  Multiple evanescent white dot syndrome (MEWDS)
  Geographic helicoid peripapillary choroidopathy (GHPC)
  Multifocal choroiditis (MFC)
  Punctate inner choroidopathy (PIC)
  Birdshot choroidopathy
  Presumed ocular histoplasmosis syndrome (POHS)
  Subretinal fibrosis and uveitis syndrome (SFU)
  Diffuse unilateral subacute neuroretinitis (DUSN)
  Retinal pigment epithelitis (Krill's disease)
  Sarcoidosis

#### 2. Clinical characteristics of a lesion

Choroiditis

Retinochoroiditis/chorioretinitis

Retinitis

Neuroretinitis

Granuloma

Mass lesions (masquerading as uveitis)

## Investigations in the diagnosis of posterior uveitis

*Ancillary investigations:* A provisional clinical diagnosis can be reached in most cases of posterior uveitis. Ancillary investigations assist in not only confirming the clinical diagnosis but also in cases of diagnostic dilemma.

*Color fundus/FFA*: Serial documentation of lesions with color fundus photographs can assist in the follow-up of the disease with treatment. FFA may be useful in confirming the activity of a choroiditis/retinitis denoted by a characteristic early hypofluorescence and late hyperfluorescence in case of active choroiditis. It can be used to detect disease sequelae such as neovascularization, capillary non-perfusion areas, and vascular staining in cases of retinal vasculitis. It can reveal a typical flower petal pattern in cystoid macular edema (CME) or as pooling of dye in late phase in VKH. FFA is most useful to detect the presence, type, and activity of choroidal neovascularization (CNV), which is a vision-threatening complication associated with many posterior uveitic entities.

*ICG*: ICG is more helpful in case of deeper choroidal lesions, CNV, and in the presence of hemorrhages. It is especially useful in detection, identification, and follow-up of entities with deeper choroidal lesions such as White dot syndromes, where FFA may not be completely confirmatory.

*OCT*: OCT is a non-contact imaging tool helpful in detecting and monitoring macular pathologies such as CME, epiretinal membrane, CNV membrane (CNVM), and macular hole. The conventional time domain OCT has a resolution of 10 microns while the newer spectral domain OCT (SD-OCT) has increased the resolution to 5 microns and gives a three-dimensional view enhancing its diagnostic potential.

*Ultrasound B scan (USG)*: It is a very useful tool, especially when the media is hazy and in cases with cataract or severe vitritis or vitreous hemorrhage. There is an indication for USG-B scan even when there is a relatively clear media in many posterior and panuveitic conditions. It helps differentiate rhegmatogenous and exudative retinal detachment based on the shifting of fluid, which is more characteristic of an exudative retinal detachment. An increased choroidal thickening can be a significant finding. It may be seen in Vogt Koyanagi Harada's disease (VKH) with significant posterior uveitic manifestations and in posterior scleritis. Normal choroidal thickness is around 1.1 mm. Presence of ‘T’ sign and/or Tenon's space widening noted in USG-B scan is pathognomonic of posterior sclerits. It is also useful to diagnose intraocular tumors masquerading as uveitis and elevated mass-like lesions such as TB subretinal abscess.

### Laboratory investigations

Tailored lab investigation relevant to the clinical entity in question is the right approach in identifying the etiology of a posterior uveitic entity. Laboratory tests are more useful in infective than in non-infective conditions. Specific tests for each entity have been described later. In cases of diagnostic dilemma, intraocular fluid evaluation for polymerase chain reaction (PCR) and antibody titers helps clinch the diagnosis.

### Systemic examination

It is extremely important that the patient be evaluated thoroughly by an internist to rule out possible associated causes of his/her uveitis and also to evaluate the laboratory test findings, as most of the uveitic entities can have systemic associations. The treatment of the patient is incomplete without simultaneous treatment of the underlying systemic condition.

## Treatment

Local and systemic steroids along with immunosuppressives in select cases are the mainstay of treatment of non-infective conditions. Infective conditions need to be treated primarily with the specific anti-infective agents along with anti-inflammatory therapy in the form of low-dose steroids. In case of infective uveitis, systemic steroids need to be initiated at least 48–72 h after start of specific anti-infective therapy and then stopped at least 1 week prior to stoppage of specific treatment.

## Infective posterior uveitis

Infective posterior uveitis is a clinical diagnosis based on characteristic fundus picture and relevant positive history. Laboratory investigations are predominantly based on antibody testing against the specific antigen and PCR testing for the particular genome. Other tests to detect associated systemic condition may be required to clinch the diagnosis. It is important to treat the underlying systemic disease along with ophthalmic treatment. Basic management approach is given in Table 2.

**Table 2:** Basic management approach – guidelines

Identify the characteristic/typical fundus picture Confirm with specific investigations Treat the primary cause with appropriate anti-infective agent Use systemic steroids as additional anti-inflammatory therapy Avoid periocular/intravitreal steroids In diagnostic dilemmas, intra-ocular fluid evaluation for antibodies or PCR for identification of genome

A comprehensive overview of the characteristic clinical appearance of common infective posterior uveitic entities and the current management approach is briefly described below.

### Ocular toxoplasmosis

Clinical diagnosis: Ocular toxoplasmosis is the most common infective cause of posterior uveitis in immunocompetent patients. Most cases of toxoplasmosis in the immunocompetent host are subclinical or benign. Prevalence is higher in tropical countries than in arid or cold areas.[3] A positive history of contact with pets such as dogs and cats,[2,3] ingestion of raw, undercooked meat, or contaminated municipal water[4] can identify the source. Transplacental transmission of *T. gondii* is the only form of human-to-human transmission.

Toxoplasmic retinochoroiditis is unilateral in 72–83% of the cases.[3] Ocular toxoplasmosis occurs from the activation of cysts deposited in or near the retina. Focal necrotizing retinitis is the characteristic lesion. Peripheral retinochoroidal scars are the most common ocular finding, occurring in 82% of the patients. However, toxoplasma has a strong predilection for the posterior pole, particularly the macular region,[2] this location occurring in more than 50% of the cases.[3]

### Congenital toxoplasmosis

**Figure 1:**
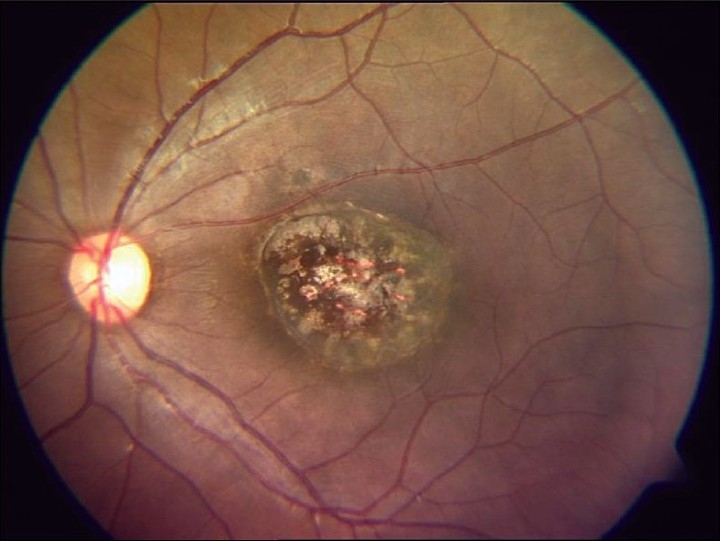
Fundus picture showing a typical punched-out macular scar of a healed congenital toxoplasmosis

Presence of an asymptomatic punched-out macular cicatricial lesion with a central necrotic zone involving the retina, choroid, and vitreous is diagnostic [Fig. 1]. Ocular infection may be the only manifestation of congenital toxoplasmosis. Ten percent of the patients have ocular lesions without clear evidence of disease in other organs.[2,3]

Recurrent lesions frequently develop at the borders of the old toxoplasma scars, so-called satellite lesions, and are called reactivation of congenital toxoplasmosis.

### Acquired toxoplasmosis

**Figure 2:**
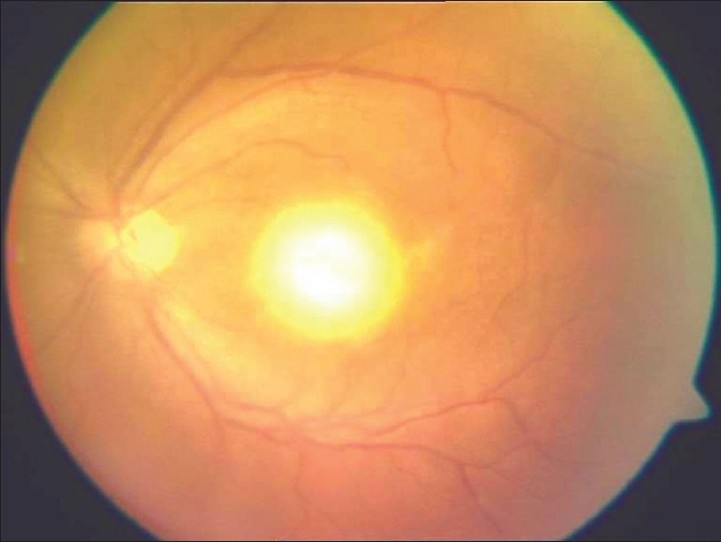
Fundus picture showing a typical “headlight in the fog appearance” in a patient with acquired toxoplasmosis

Patients with ocular toxoplasmosis with macular involvement usually present with diminished vision and/or floaters. “Headlight in the fog” appearance of a focal necrotizing retinochoroiditis with overlying vitritis is the characteristic [Fig. 2]. Classically, the initial lesion starts in the superficial retina, gradually involving the full-thickness retina, adjacent choroid, vitreous, and even sclera. A yellowish white or grey exudative lesion is seen with ill-defined borders because of the surrounding area of retinal edema. The size of the lesion varies from a fraction of the disc to about two quadrants of the retina. Adjacent choroiditis, hemorrhage, and vitreitis may be seen. It is relatively asymptomatic in peripheral lesions,[3] seen in 70–90%, and patients may present with only floaters.

Vascular involvement may be noted close to the active lesion or in the distant retina and can present as a diffuse or segmental vasculitis. This is produced by the antigen–antibody complex deposition and/or localized mononuclear cell infiltrates in the vessel wall. Although phlebitis is common, arterial involvement is also seen. Kyrieleis arterialitis (exudates or periarterial plaques) can also be seen.[2]

The healed scar has well-defined borders around the central retinochoroidal atrophy. Uncommon presentations include associated serous macular detachment,[5] retinal vasculitis, neuroretinitis[6] with papillitis, disc hemorrhages with venous engorgement, and macular star. Anterior uveitis is a complication of the retinochoroiditis and the presence of the parasite in the anterior segment has been demonstrated in immunocompromised patients.[3]

In immunocompromised individuals, as in acquired immune deficiency syndrome (AIDS), punctate outer retinal toxoplasmosis or necrotizing retinitis lesions mimicking viral retinitis may be seen.

Secondary glaucoma is the most common complication. Others include cataract, vitreous hemorrhage, retinal detachment, CNVM, CME, vascular occlusions, and optic atrophy.

Confirmation of diagnosis: Diagnosis is mainly clinical and ancillary investigations like FFA, ICG,[7] and OCT are complimentary.[3] Although detection of toxoplasma-specific antibodies in serum is useful in atypical cases, high titers of positive toxoplasma antibodies in the normal human population may complicate results. Serum antitoxoplasma antibody titers can be determined by several techniques such as Sabin–Feldman dye test (Gold standard), complement fixation test, hemagglutination test, immunofluorescence antibody test, enzyme-linked immunosorbent assay (ELISA), immunoblotting, and immunosorbent agglutination assay, but ELISA is the most common serological test employed.

Antibody detection and characterization differentiates recently acquired and chronic infections[3] (immunoglobulin [Ig] M and IgG respectively). Acute systemic toxoplasmosis has traditionally been diagnosed by seroconversion. Anti-Toxoplasma IgG titers present a 4-fold increase that peak 6–8 weeks following infection, then decline over the next 2 years, but remain detectable for life.

Anti-Toxoplasma IgM appears in the first week of the infection and then declines in the next few months. Antibody titers in the serum do not always correlate with ocular disease and hence management has to be based on the clinical diagnosis. Anti-Toxoplasma antibodies may be very low and should be tested in undiluted (1:1) samples if possible. It is to be noted that raised IgG antibody titres, which may be seen in other uveitic conditions, should not form the basis for treatment with anti-Toxoplasma drugs if there is no clinical evidence suggestive of active ocular toxoplasmosis.

PCR is an important tool in the diagnosis of ocular toxoplasmosis, especially in cases of equivocal serology. Various groups have compared PCR and Goldman–Witmer (GW) co-efficient analysis and have been found to be of equal utility.

In case of diagnostic dilemma, aqueous or vitreous samples may be evaluated for the presence of Toxoplasma DNA sequences, using this technique. Antibodies titers are measured in aqueous humor and serum and GW co-efficient is calculated.

A combination of PCR testing and GW co-efficient of antibody titers in aqueous or vitreous[8–10] has a high degree of specificity and sensitivity. Analysis of IgG, IgM, and IgA increases the sensitivity of the aqueous humor study[2,9–11] and helps rule out viral etiology, especially in immunocompromised individuals.

**Treatment:** An ideal combination that destroys tissue cysts and prevents recurrence has not been found[2,12–14] as current therapies are targeted only on trophozoites. Pyrimethamine(100 mg-1st day, 75 mg-2^nd^ day, 50 mg-3^rd^ day, followed by 25 mg once daily) and Sulfadiazine (4 g daily-divided 6^th^ hourly) for 4–6 weeks, is the most effective combination that works synergistically. The other drugs used in the treatment of toxoplasmosis are given in Table 3.

**Table 3:** Other anti-Toxoplasmic drugs

Pyrimethamine (100 mg-1^st^ day, 75 mg-2^nd^ day, 50 mg-3^rd^ day, followed by 25 mg once daily) + Sulfadiazine (4 g daily-divided 6^th^ hourly) for 4–6 weeks Clindamycin[14] (300 mg 6th hourly-maximum dose:1.8 gm/day) for 6 weeks Trimethoprim+sulphamethoxazol–DS tab–160 mg/800 mg–one tab twice daily for 6 weeks Spiramycin–2 g/day in two divided doses Azithromycin–loading dose 1 G-1^st^ day, followed by 500 mg once daily for 3 weeks Atovaquone[13]–750 mg every 6 h for 4–6 weeks

Treatment regimen consisting of a sulfonamide and a non-sulfonamide with systemic steroids and folic acid supplements is preferred. In patients with sulfa allergy, clindamycin and azithromycin are suitable alternatives. Topical steroids and cycloplegics are used to treat associated anterior uveitis. Immunocompromised individuals require long-term prophylaxis till improvement in immune status of the individual even after resolution of lesions.[3] It is important to rule out associated central nervous system involvement in patients with AIDS.

Modifications in the treatment regimen in various special situations such as pregnancy and in neonates is given in Table 4.

**Table 4:** Anti-Toxoplasma therapy in special situations

Pregnancy	I trimester – Spiramycin + Sulfadiazine
	II trimester (>14 weeks) – Spiramycin + sulfadiazine + pyrimethamine + folinic acid
	III trimester – Spiramycin + pyrimethamine + folinic acid
Newborn	Pyrimethamine, sulfadiazine, and folinic acid
Mother	Reduces the likelihood of congenital transmission

Toxoplasmic CNVM has been treated effectively with verteporfin photodynamic therapy.[15,16] Pars plana vitrectomy is performed to treat persistent vitreous opacities or vitreoretinal traction. A newer fluoroquinolone, trovafloxacin, has potent anti-Toxoplasma activity and appears promising. Sobrin *et al*.[17] reported favorable response in six patients of toxoplasmic retinochoroiditis with intravitreal clindamycin with/without pars plana vitrectomy.

### Ocular toxocariasis

**Clinical diagnosis:** Toxocariasis is an infection caused by the accidental ingestion of larvae of the dog roundworm *Toxocara canis* or the cat roundworm *Toxocara cati*. Children who have pica and are in close contact with puppies are particularly vulnerable. Ocular toxocariasis is diagnosed based on a positive history of contact with pets and suggestive ocular findings. Risk of human infection is higher in children with pica who may ingest contaminated soil or meat. Infections in humans, an end host, result in a focal granulomatous reaction in many organs, including the eye.

Various clinical manifestations according to the decreasing preference of the parasite are:

1. Granuloma in the peripheral retina and vitreous
2. Posterior pole granuloma
3. Chronic endophthalmitis
4. Optic nerve involvement
5. Anterior segment involvement

**Figure 3:**
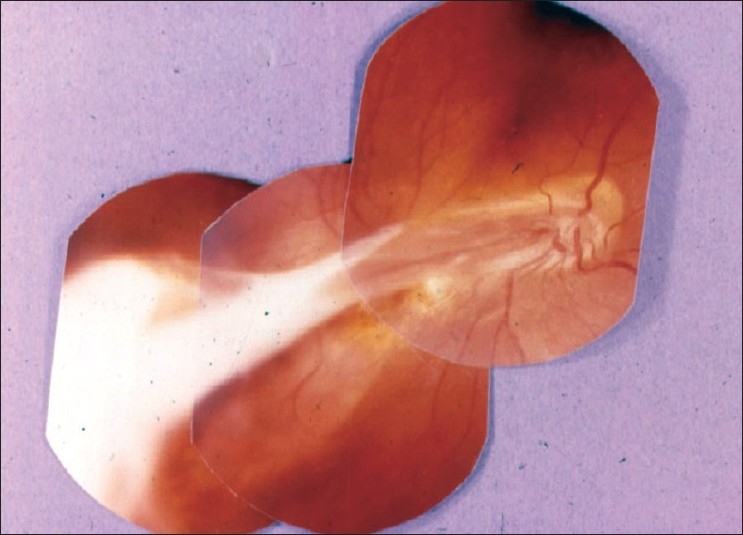
Fundus picture showing a toxocara granuloma

Presence of a posterior pole or a peripheral granuloma [Fig. 3] with tractional retinal detachment or a chronic endophthalmitis-like picture[18] impairing visualisation is typical. Early lesions may be poorly visualized because of intense vitreous haze. Longstanding masses may have substantial secondary atrophy and hyperplasia of the retinal pigment epithelium (RPE). Whitish/grayish-white granulomas of various sizes may rarely be seen in juxta papillary and subfoveal locations. Inferiorly located lesions can sometimes be mistaken for parsplanitis. Dead larvae are sometimes seen as a dark grey area within the whitish mass at the posterior pole. CNVM and a subretinal toxocara granuloma have to be ruled out.[19]

**Confirmation of diagnosis:** Diagnosis is mainly clinical, although ELISA with Toxocara excretory-secretory antigen (TES-Ag) has been shown to be highly specific for toxocara infection. An increase of anti–TES-Ag IgE level indicates acute toxocara infection or progressive inflammation. An increase in the IgG level confirms a past or present infection with minimum inflammation. Toxocara GW co-efficient analysis from aqueous and serum can be of value when diagnosing patients with posterior focal lesions or vitritis of unknown etiology.[20] USG/computerized tomography (CT) findings have additional value.

### Differential diagnosis:

- Retinoblastoma may mimic toxocariasis with calcification
- Rule out endophthalmitis, severe pars planitis, juvenile idiopathic arthritis-associated uveitis, Coats' disease, primary hypertrophic proliferative vitreopathy, familial exudative vitreoretinopathy, and late stages of retinopathy of prematurity.

**Treatment:** No large case control trial as yet has compared anti-helminthic therapy for ocular toxocariasis against observation alone. Systemic steroids is the mainstay and it is important to monitor its side effects, especially growth retardation in children. Surgical treatment such as pars plana vitrectomy, cryopexy, and laser photocoagulation has been used to treat complications. Vitrectomy may be beneficial for patients with endophthalmitis, unrelieved vitreoretinal traction with retinal detachment,[1] and also as an optical indication.

### Tubercular posterior uveitis

Ocular TB can be primary, where the eye is the initial site of entry or secondary, where organisms spread to the eye hematogenously; this type includes tuberculous uveitis. Hypersensitivity reaction to tuberculous protein can also cause retinal vasculitis. A study from our center noted that only 1.39% patients with systemic TB had tubercular uveitis.

**Clinical diagnosis:** The most common presentation of tuberculous uveitis is of disseminated choroiditis.[21] Choroidal tubercles may be one of the earliest signs of disseminated disease. The lesions may vary from few numbers to several hundred. The lesions range from 0.5 to 3.0 mm in diameter and may vary in size and elevation within the same eye. They are deep in the choroid, appear yellow, white, or gray, and are fairly well circumscribed. In the vast majority of cases, the lesions present in the posterior pole.

**Figure 4:**
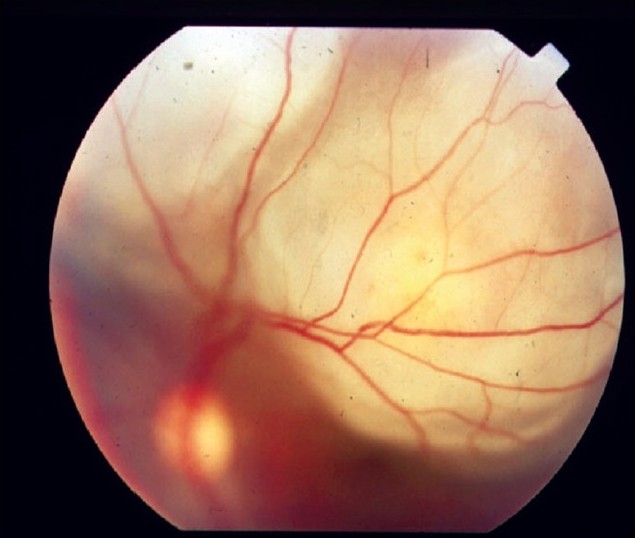
Fundus picture showing a tuberculous subretinal abscess

The next most common presentation is a single tubercle, also termed focal choroiditis,[22] which can occur at the posterior pole. A single choroidal mass is the characteristic feature on presentation, although multiple choroidal tubercles can be seen in cases of miliary tubercles and in immunosuppressed individuals. A large tubercle may measure up to 4.0 mm in diameter; however, choroidal masses up to 14 mm in diameter have been reported. The mass is typically elevated and may be accompanied by an overlying serous retinal detachment[23] [Fig. 4]. Subretinal abscess is formed progressively from a choroidal tubercle, which can be single or multiple. Diagnosis is based on clinical features, suggestive of systemic findings and supportive investigations.

Solitary tubercular choroidal granuloma[24,25] can be misdiagnosed as choroidal melanoma due to multiplicity of clinical findings, which also causes diagnostic delay. Other presentations include serpiginous-like choroiditis,[26] retinal vasculitis,[27] intermediate uveitis, panuveitis, and neuroretinitis.

**Confirmation of diagnosis:** Angiography confirms activity in choroiditis[28] and reveals a classical ring of fire appearance in the subretinal abscess/choroidal granuloma. Retinal vasculitis can present with neovascularization and capillary non-perfusion areas due to inflammatory vessel obstruction, which may need prophylactic laser panretinal photocoagulation. OCT scans through areas of suspected granuloma can be helpful in differentiating choroidal granulomas from other non-inflammatory conditions, such as central serous chorioretinopathy (CSR), exudative age-related macular degeneration, and choroidal tumors mimicking choroidal granuloma. In TB granuloma, OCT[29] reveals an area of localized adhesion between the choriocapillaris–RPE layer and overlying the neurosensory retina (“contact” sign), possibly due to inflammatory adhesions overlying the granuloma that cause the neurosensory retina to stick to the RPE at that point. Inflammatory cells appear as increased reflectivity in the deeper retinal layers over the granuloma. These features, unique in TB and other inflammatory conditions, are unusual in non-inflammatory lesions. USG-B scan helps rule out tumors in large mass like subretinal abscesses.

Associated systemic latent/manifest TB confirms diagnosis. Mandatory investigations include hemogram, erythrocyte sedimentation rate (ESR), Mantoux test, and radiological imaging such as chest X-ray/CT scan.

It is important to note that tuberculin test can be false positive in patients hailing from endemic countries like India and is also affected by previous BCG vaccination.

Newer tests based on gamma interferon assays such as the Quantiferon TB-Gold test are promising.[30] Others include BACTEC MGIT 960 system, MB/BacT system, and the ESP II culture system.

Histopathological and/or microbiological confirmation of mycobacterium infection, especially by PCR, from intraocular specimens is diagnostic. IS6I I0 primer-based PCR is widely used for detection of the *M. tuberculosis* complex. However, nested PCR technique employing the MPB64 gene is 10,000 times more sensitive and 100% specific and is used in cases of doubt.[31–33] Real-time PCR technology can differentiate commensals and contaminants from infecting microbes. Dot-blot hybridization of the PCR product by 32 P-labeled specific probes improves sensitivity.[34]

**NOTE:** ELISA and PCR testing for TB on serum are not useful, especially in endemic regions like India, and should not form the basis of diagnosis of intraocular TB.

**Treatment:** It is imperative that anti-tuberculous therapy (ATT) be initiated under care of an internist once tuberculous etiology is confirmed. Concomitant systemic steroids for 4–6 weeks have a protective effect against tissue damage from delayed type of hypersensitivity (DTH). Use of corticosteroids alone should be avoided as it promotes multiplication of bacilli and can lead to panophthalmitis. Guidelines described by Gupta *et al*.[35] can form the basis for diagnosis and management of ocular TB. Successful medical management of subretinal tubercular granuloma have also been described,[36] where authors have concluded that once the diagnosis of presumed or confirmed TB is established, surgical intervention should be avoided and successful resolution of lesions can be noted with ATT and steroids alone. Surgery is an option mainly for complications.[37]

### What's new?

- Newer quinolones and rifamycins like rifabutin and macrolides are part of ATT regimes, especially in multidrug-resistant TB.
- Usefulness of Retinalamine in patients with TB chorioretinitis is being evaluated.[38]

### Viral retinitis

A retinitis should evoke a suspicion of a viral infection, which is usually caused by *Herpes simplex*, *Varicella zoster,* and CMV. Other rare causes include chikungunya and rubella viruses.

Herpetic eye disease is among the most common causes of infectious uveitis. It may affect healthy as well as immunocompromised hosts, although its clinical presentation varies accordingly. Posterior uveitis caused by herpes viruses may appear as part of herpetic disease elsewhere (skin, brain, anterior segment of the eye) or as an isolated finding. Most forms of herpetic posterior uveitis are acute and fulminant, often resulting in serious complications such as retinal detachment and proliferative vitreoretinopathy.

Presentation of herpetic infections varies based on the immune status of the individual. Associated systemic herpetic manifestations, a past history of chicken pox, or signs and symptoms of an immunocompromised state may be diagnostic clues.

**Clinical diagnosis:** Acute retinal necrosis (ARN) is the classical presentation of herpetic viruses. Characteristic clinical triad of moderate to severe vitritis, arteritis, and periphlebitis and confluent peripheral retinal necrosis is diagnostic of ARN, which can present as a panuveitis. Although the posterior pole is not typically affected early in the disease process, it can also be involved primarily in necrotizing herpetic retinopathies.

**Figure 5:**
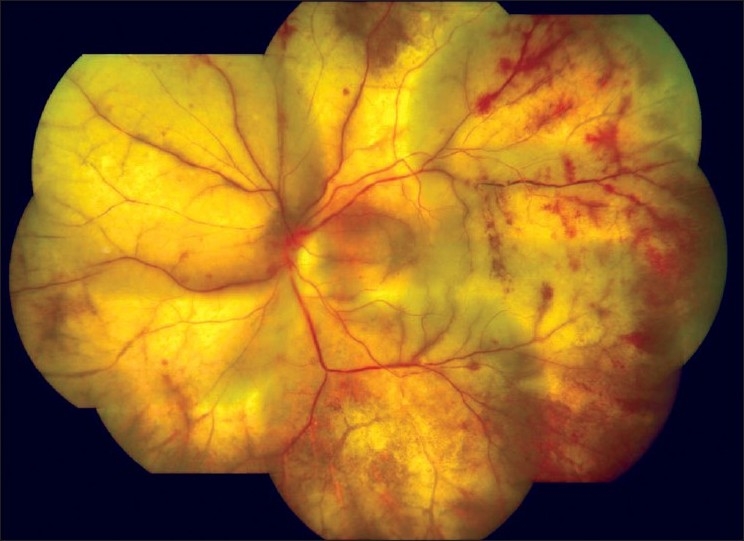
Fundus picture showing the classical “cracked mud appearance” in progressive outer retinal necrosis

*Progressive outer retinal necrosis (PORN)*: Necrotizing retinitis, confluent areas of outer retinal whitening with minimal vitritis involving the posterior pole and sparing of retinal vessels at the early stage, the typical “cracked mud appearance”[Fig. 5] is virtually diagnostic.[39] Although ARN and PORN are considered to be two different entities, they are considered essentially as different manifestations of the same disease and the varied presentations occurring due to differences in the immune status of the host. PORN is frequently bilateral, occurring exclusively in immunocompromised state, such as in patients with HIV infection,[39] and is associated with rapid development of rhegmatogenous retinal detachment or optic atrophy.

Viral posterior uveitis can also rarely present as patchy, single, or multiple retinitis patches, which includes acute varicella retinitis/choroiditis seen in children as well as chronic choroiditis or non-necrotizing retinal vasculitis in adults.

Chikungunya retinitis[40–42] mimics herpetic or CMV retinitis and is diagnosed based on a history of ailment with chikungunya disease and detection of specific antibodies to the virus from serum or intraocular fluid. It is often self-limiting and, although not specific, acyclovir or intravitreal ganciclovir have been found to be beneficial.

**Figure 6:**
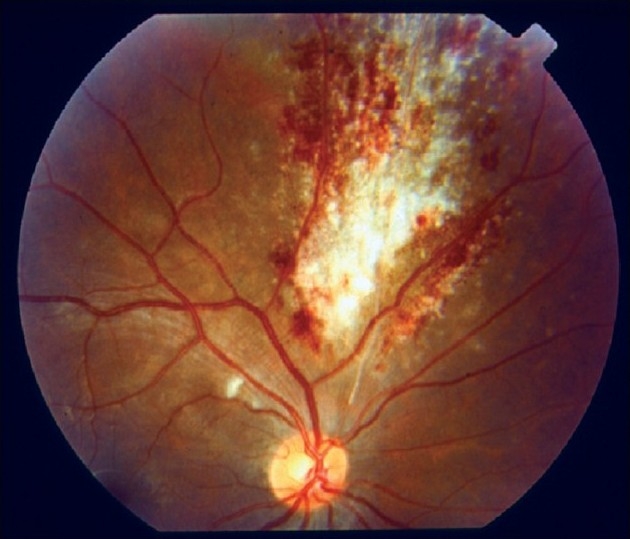
Fundus picture showing a typical “pizza pie appearance” in a patient with cytomegalovirus retinitis

CMV retinitis, usually seen in immunocompromised states such as in AIDS or post organ transplant patients on immunosuppressives, has a characteristic granular or pizza pie appearance [Fig. 6] and a typical brushfire pattern of spread along the blood vessels.

Although rubella virus, which is part of the ‘TORCH’ complex, can also cause ocular lesions, rubella retinopathy can be clearly differentiated from retinitis due to herpetic viruses as it has a characteristic salt-pepper appearance and can mimic retinitis pigmentosa.

**Treatment:** Long-term systemic antiviral drugs such as acyclovir or valaciclovir are the treatment of choice in case of herpetic retinitis. Aciclovir is very effective against HSV and VZV. Treatment with acyclovir reduces infection of the fellow eye from 70 to 13% in the first year.[2]

The dosage is 15 mg/kg body weight in three doses for 7–21 days. Then, 2–4 g daily is recommended for a further 4–6 weeks.[2] Usually, intravenous acyclovir 500 mg 8^th^ hourly for 1–2 weeks followed by oral acyclovir 800 mg 5-times daily is recommended with additional low-dose systemic steroids. Alternatively, oral valaciclovir, which has a better bioavailability, can be given as 1 g three times daily. Antivirals may be continued for almost 3–6 months in some cases. Renal function needs to be monitored if antivirals are administered on a long-term basis, especially in extremes of age. Prophylactic laser barrage adjacent to retinitis lesions is known to prevent retinal detachment, although there is no randomized study that validates the use of laser barrage for preventing a subsequent retinal detachment. Early vitrectomy as an option has been advocated but also needs to be tested in a randomized fashion. In resistant cases, alternatives include a combination of systemic and intraocular antiviral therapy with foscarnet and ganciclovir.[43]

Intravenous ganciclovir-5–7 mg/kg/day in two divided doses for 2 week-induction dose followed by once daily-maintenance dose till complete resolution of lesions and improvement of immune status is the treatment of choice in CMV retinitis. Intravitreal injections of either ganciclovir or foscarnet may be considered, especially in initial cases where the macula is threatened. It can be a way to get the greatest concentration of drug to the affected area immediately.

Alternatively, oral valganciclovir-900 mg BD as induction and 900 mg OD as maintenance dose has an additional advantage of being a non-parenteral mode of treatment, avoiding complications related to indwelling catheters, especially in immunocompromised individuals. Appropriate management of underlying systemic disease under care of an internist promotes early resolution of ocular lesions.

**NOTE:** Necrotizing retinopathy due to toxoplasma closely mimics herpetic retinitis, especially in immunocompromised patients.

CMV retinitis can also involve the posterior pole initially and, in early stages, can mimic cotton wool spots of HIV retinopathy.

### Ocular syphilis

Ocular syphilis can mimic any of the uveitic entities, and has to be ruled out especially in any case of infective uveitis. It is the most common intraocular bacterial infection and is re-emerging in varied forms, especially with the advent of AIDS.[44] About 1–2% of HIV-positive patients are found to have ocular syphilis.

Clinical diagnosis: Syphilis is called the great imitator as it can present as chorioretinitis, neuroretinitis, salt-pepper retinopathy, optic neuritis, papilloedema, and optic perineuritis.^1^ Syphilis can present with placoid choroidal lesions and can mimic APMPPE. An unusual manifestation of syphilis is acute necrotizing retinopathy, which mimics ARN. Syphilitic uveitis can be the first presentation of the systemic disease[45] in both immunocompetent and immunocompromised individuals. In HIV-positive patients, ocular syphilis is more closely associated with neurological abnormalities.

Confirmation of diagnosis: Laboratory investigations such as venereal disease research laboratory test, rapid plasma reagin test, and treponema pallidum hemagglutination tests are used. Confirmatory tests include fluorescent treponema antibody agglutination (FTA-ABs) test and dark ground microscopy. A specific treponemal test such as FTA-ABs can be used as an initial test for a syphilis screen as it is more reliable and has low false positivity. Patients often show abnormal cerebrospinal fluid findings. Diagnosis is very challenging as up to 38% of HIV-positive patients can be seronegative despite active syphilitic disease.[46]

**Treatment:** Treatment of ocular syphilis is similar to that of neurosyphilis. Long-acting penicillin is the treatment of choice.

**Figure 7:**
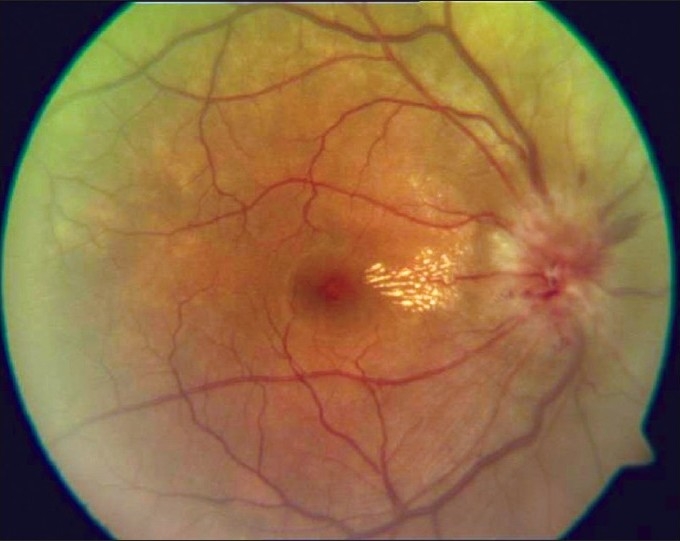
Fundus picture showing disc edema and macular exudates characteristic of a neuroretinitis

**Neuroretinitis:** Involvement of the optic nerve head along with retinitis and macular star constitutes the typical appearance of a neuroretinitis [Fig. 7]. It can be caused due to toxoplasma, TB, syphilis, and other non-infective conditions such as collagen vascular diseases, and is treated accordingly.

Cat-scratch disease (CSD) caused due to Bartonella is an important cause of neuroretinitis. It can also present with acute macular neuronopathy.[47] Doxycycline-100 mg is the treatment of choice. Multifocal chorioretinal lesions associated with *B. hensalae* can be atypical ophthalmic manifestations of CSD, which may occur in immunosuppressed patients.[48] Recognition and appropriate treatment of underlying disease gives better visual outcomes.

### DUSN

DUSN, a rare entity caused by a worm in the eye, is usually unilateral. Clinical visualization of subretinal nematode makes the diagnosis obvious. Live worm, if detected, can be destroyed by direct laser application. Worm and its by-products can cause severe inflammation affecting the optic nerve head and retina and needs to be treated with high-dose systemic steroids. If a worm is not found clinically, appearance of sub-RPE serpiginous tract in the inferotemporal retina, peripheral RPE hypopigmentation, good clinical response to anti-helminthics, and abnormal ERG supports the diagnosis.[49] Treatment with albendazole is beneficial.

### Intraocular cysticercosis

Cysticercus cellulose cyst, found inside the eye, can cause severe inflammatory reaction.[50,51] When visualization is impaired, USG-B scan is diagnostic. Neurological association needs to be ruled out. Treatment is with steroids and anti-helminthic therapy.[50] Removal of the cyst is necessary in most cases.

### Non-infective posterior uveitis

Common non-infective posterior uveitic entities include the “WDS” and are named according to their clinical appearance and behavior. FFA and ICG are virtually diagnostic in WDS. Laboratory investigations are not routinely necessary for their diagnosis but are essential for monitoring therapy-related side effects. A significant percentage of patients with WDS have a prodromal viral-like illness that triggers the onset of the ocular condition and may be self-limiting, although they need to be followed-up closely for complications such as scarring and CNVM. Patients with WDS present with sudden blurring of vision associated with photopsia, floaters, scotomata, and metamorphopsia. An approach to a case of inflammatory posterior uveitis is dipicted in [Table 5].

**Table 5:** Basic management approach to a case of non-infective posterior uveitis

Identify the clinical entity based on characteristic clinical feature
Rule out infective causes
Systemic steroids are the mainstay of therapy
Use intravenous methyl prednisolone therapy in vision-threatening lesions
Use immunosuppressives, with caution, in recalcitrant cases
Monitor side effects of treatment

The comparative clinical characteristics and investigative findings of various WDS are given below [Tables 6–8].

**Table 6:** Comparative characteristics of clinical presentations of white dot syndrome[52]

	APMPPE	Birdshot	PIC	MEWDS	MFC	GHPC	POHS
Age	Young (20–40) Rarely-children	Middle-aged (40–60)	Middle aged (myopes)	Young (20–40) myopes	Myopic (20–60)	Variable (30–60)	Middle aged
Sex	M=F	F>M	F>M	F>M	F>M	M>F	M=F
Laterality	Bilateral, asymmetric	Bilateral	Bilateral	Unilateral	Bilateral; asymmetric	Bilateral; asymmetric	Bilateral
Viral illness	+	-	+	+	+/-	-	+/-
Onset	Abrupt	Insidious	Abrupt	Abrupt	Insidious	Variable	Abrupt
Duration	Weeks–months	Chronic	Weeks–months	Weeks–months	Chronic	Chronic	Chronic
Recurrence	Rare	Recurrent	Recurrent	Rare	Recurrent	Recurrent	Rare
Vitritis	Mild	Moderate with disc edema, CME	Absent	Mild	Moderate and anterior uveitis	Mild	Absent/mild
ERG/EOG	Abnormal EOG	Abnormal ERG	Abnormal	Abnormal ERG	Abnormal ERG	Normal	Abnormal
HLA	B7, DR2	A29	-	-	-	B7	HLA-DR2 HLA-B7
Fundus - active	Multifocal, flat gray-white placoid lesions primarily-posterior pole at the level of RPE and chorio capillaries	Multiple depigmented yellow-white patches scattered throughout fundus in the postequatorial region. These lesions radiate from optic nerve and follow larger choroidal vessels	Multiple, discrete, flat, yellow, round lesion (50–300 microns) at the level of RPE and inner choroid. Concentrated at posterior pole	Multiple small (100–200 μ), round, slightly indistinct, white/yellow-white spots distributed over posterior fundus, especially at perifoveal and peripapillary regions at the level of RPE	Multiple yellow or gray lesions at the level of choroid and RPE. Mid periphery (50–100 μ)	Macular, peripapillary or ampigenous -irregular, gray-white or cream-yellow subretinal infiltrates at the level of the choriocapillaries and RPE -snake-like pattern	Peripapillary atrophy, atrophic chorioretinal lesions, CNV, punched out yellow lesions Linear streak-smidperiphery
Fundus- healed	RPE clumping and hyperpigmentation	Lesions have a hyperpigmented edge but are frequently hypopigmented in the center		Heals rarely by scarring	Punched-out atrophic scars that develop pigmentation over time	Heals from center towards periphery	Scars
Pathogenesis	DTH	Auto immune	-	?Hormonal	-	Idiopathic/ ?infective	-

Wks–Weeks, DTH–Delayed type of hypersensitivity

**Table 7:** Comparative characteristics of FFA and ICG features of white dot syndrome[52]

	APMPPE	Birdshot	PIC	MEWDS	MFC	GHPC	POHS
FFA active	Early hypofluorescence and late hyperfluorescence	Mild hyperfluorescence and staining in late phase	Early hypofluorescence and late hyperfluorescence	Early patchy punctate hyperfluorescence with late deep staining of RPE and peripapillary area. Leakage from optic disk and retinal capillaries. Early fluorescence-wreathlike pattern. Choroidal background fluorescence between lesions is normal	Early hypofluorescence and late hyperfluorescence	Early hypofluorescence and late hyperfluorescence. Choroidal vessels are easily seen	Early hypofluorescence and late hyperfluorescence. Confirms CNVM
FFA inactive	Window defects	Window defects	Window defects	Window defects	Window defects. Early hyperfluorescence and late staining	Window defects	Window defects
ICG-active	Marked choroidal hyperfluorescence in both early and late phase. Large choroidal vessels seen	-	CNVM reveals hyperfluorescence	Multiple hypofluorescent spots in the posterior pole and hyperfluorescence around optic nerve head, especially in patients with enlarged blind spots	Hypofluorescent CNVM reveals hyperfluorescence	-	-
ICG-active	Choroidal hypofluorescence	-	-	Hypofluorescent spots persist until patient recovers	Hypofluorescent.	-	-
ERG/VEP	-	unilateral negative ERG	-	markedly reduced a wave and early receptor potential amplitudes suggesting primary involvement of RPE	-	-	
Visual fields	-			Enlargement of blind spot No correlation of field defects with lesions			

**Table 8:** Clue to diagnosis of white dot syndrome

Characteristics of lesions	Consider
Subtle lesions	MEWDS
Prominent lesions	MFC
Placoid lesions	APMPPE if discrete and if they are coalesced, consider ampigenous or serpiginous choroiditis
Discrete lesions	MEWDS, DUSN, MFC, Birdshot chorioretinopathy

### Overview of clinical and diagnostic features of WDS

**Figure 8:**
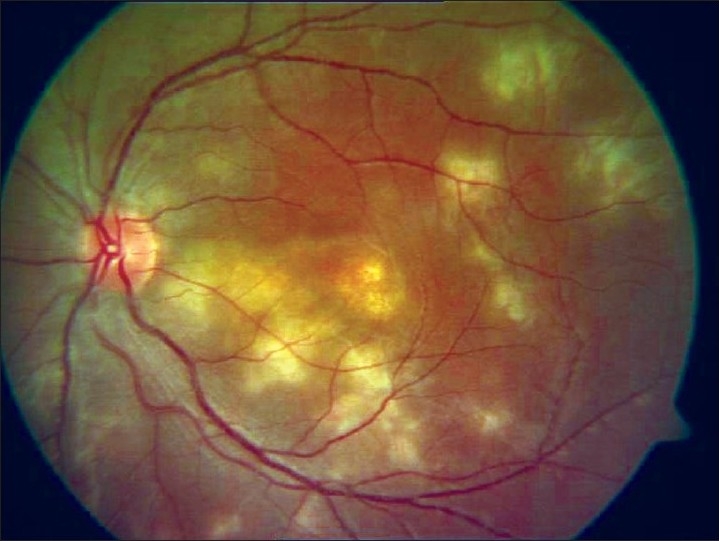
Fundus picture showing placoid lesions of acute posterior multifocal placoid pigment epitheliopathy

**APMPPE** [Fig. 8]: It is an inflammatory retinal/choroidal disease characterized by sudden loss of vision caused by the sudden appearance of multiple yellow-white, flat inflammatory lesions lying deep within the sensory retina, most notably at the level of the RPE and the choriocapillaries.[1] Atrophy and RPE scarring[52] results in poor visual acuity. New lesions may be observed in the peripheral fundus for up to 3 weeks and tend to be more linear. Additional findings include disc edema, keratic precipitates, retinal vasculitis, neurosensory detachments, and venous occlusions.[1] OCT findings in conjunction with FFA and microperimetry are useful.[53]

APMPPE has a self-limited clinical course, with spontaneous recovery of vision in most cases. Of those eyes that are affected, 90% of them typically achieve a visual acuity of more than 20/25. In rare cases, recurrences may occur within 6 months of the initial episode, thereby giving a less-favorable prognosis. Corticosteroids and cytotoxic drugs are indicated, especially in cases of associated cerebral vasculitis.[54] CNV is a rare complication.

### NOTE:[52,54,55]

- APMPPE mimics non-inflammatory conditions like multifocal choriocapillary infarcts due to hypertension, toxemia of pregnancy, and disseminated intravascular coagulation.[52]
- Rule out associated cerebral vasculitis.[55]

**MEWDS:** MEWDS is a rare disorder of unknown etiology characterized by the presence of white lesions deep in the outer retina or at the level of the RPE. It can also present with optic disc edema, mild vitritis, panuveitis, diffuse choroidal thickening, and a relative afferent pupillary defect. Newly recognized angiographic features termed dots and spots, which varied in size and location in the fundus, have been reported. Small dots were in the inner retina or at the level of the RPE, and larger spots were more external in the subpigment epithelial area. Presence of white dots around the nerve is rare and field defect persists even after lesions disappear.[51]

A viral prodrome is known in 50% of the patients, and the multifocal nature of the disease, a viral prodrome, is thought by many. Patients also present with acute, painless, unilateral loss of vision.

It can be distinguished from other WDSs by its distinct morphology, associated macular granularity, transient nature, characteristic angiographic appearance, unilaterality, self-limiting course, lack of significant sequelea, absence of associated systemic involvement, rapid recovery, and excellent visual outcome.

MEWDS is a self-limited disease, with almost all patients regaining good visual acuity within 3–9 weeks. The lesions disappear without scarring and photopsias and scotomata gradually resolve. Occasionally, patients with MEWDS may have persistent blind spot enlargement. Although uncommon, recurrences can occur. However, the prognosis is fairly good for these patients. A rare complication of MEWDS is CNV, which may require laser photocoagulation.

### NOTE:

- Careful evaluation by slit lamp biomicroscopy[1] is a must.
- Characteristic finding of MEWDS is foveal granularity.[56,57]
- Multifocal ERG helps differentiate MEWDS from other blind spot enlarging conditions.[58,59]

### Serpiginous choroiditis[60] (GHPC)

It is a rare, chronic, progressive, and recurrent bilateral inflammatory disease involving the RPE, the choriocapillaries, and the choroid. It is characterized acutely by irregular, gray-white or cream-yellow subretinal infiltrates at the level of the choriocapillaries and the RPE. Based on clinical presentation, it can be classified into (1) peripapillary, (2) macular, and (3) ampiginous types. The clinical course, regardless of the presentation, is progressive, with multiple recurrences leading to potentially significant visual loss.

**Figure 9:**
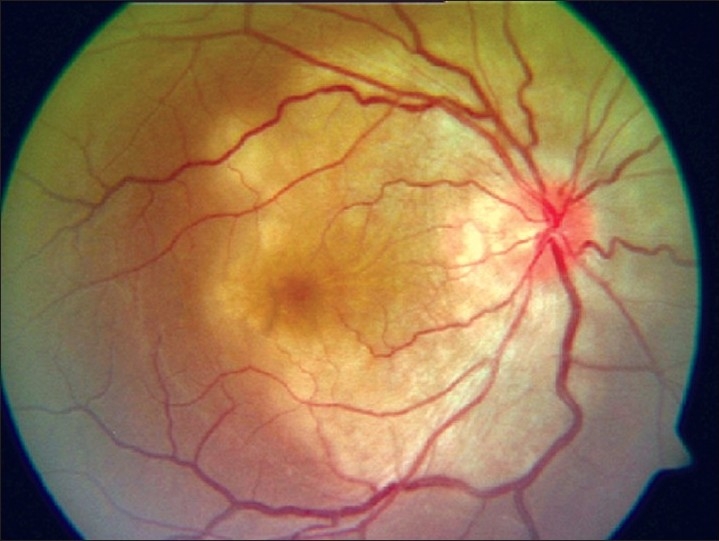
Fundus picture showing active geographic helicoid peripapillary choroidopathy

Patients present with unilateral or bilateral visual loss when the macula is involved and they may also notice photopsias and scotomata. The anterior segment usually appears quiet, although a non-granulomatous anterior uveitis has been described. Gray-white lesions are noted at the level of the RPE. Active lesions are usually found at the border of inactive lesions and appear in an interlocking polygonal pattern that spreads out the periphery from the optic nerve [Fig. 9]. Macular involvement is common. Mild vitreous and anterior chamber inflammation is observed in one-third of the cases.

Ampigenous choroiditis mimics placoid lesions of APMPPE and coalesced lesions of GHPC. Persistent placoid maculopathy is a resistant form of serpiginous choroidopathy and resembles macular GHPC, but differs in its clinical course and effect on visual acuity as a majority of the eyes develop CNVM,[61,62] resulting in central vision loss. Ten to 12% can have macular involvement alone.[63] Mild vitreous and anterior chamber inflammation is observed in one-third of the cases. Histopathology reveals lymphocytic infiltration in the affected choroid and presence of fibroglial tissue surrounding the Bruch membrane.

### Note:

- To rule out tuberculous etiology in patients with serpiginous-like choroiditis.
- PCR-proven viral etiology has also been implicated.[63]
- Toxoplasma infection presenting like serpiginous choroiditis has also been reported.[64]

### MFC

Bilateral involvement is present in approximately 66–79% of the patients.[65] Optic disc edema, rarely, peripapillary scarring, and prominent linear chorioretinal streaks may also be present. Patient may also present with CME and CNVM.[1]

### PIC[53,66,67]

PIC is an inflammatory multifocal chorioretinopathy of unknown etiology. It presents with an acute bilateral loss of vision, photopsias, and scotomata. The anterior segment is quiet. The vitreous is clear without inflammatory cells. The lack of vitreous inflammation is a hallmark of PIC and the presence of vitritis should suggest a different diagnosis.[53]

No treatment is advised for the majority of patients without CNVM or subretinal fibrosis, which usually occurs within the first year.[66] Patients without CNVM have excellent visual outcomes.[1]

### Note:

PIC occurs in myopic women.

Absence of vitritis–Hallmark of PIC

Presence of vitritis–Suggests a differential diagnosis

### SFU

Progressive subretinal fibrosis with multifocal lesions of the RPE and choroid can be seen in association with PIC and recurrent MFC. Although steroids may benefit initially, progressive fibrotic subretinal lesions leads to severe and permanent visual loss.[67] Fibrosis is predominantly at areas of previous inflammatory lesions and a turbid SRF that overlies the lesions is also noted. SFU is also seen in other inflammatory and non-inflammatory conditions,[68] such as late stage of serpiginous choroiditis, SLE-associated CSR, and onchocerciasis.[1] Infliximab has been tried with good results.[69]

### Uncommon WDS in India

Birdshot chorioretinopathy (BCR)–vitiliginous choroiditis: Research criteria for its diagnosis have been formulated[70] and more than 90% of the patients with BCR are HLA-A29 positive.[71,72] Associated non-granulomatous uveitis is seen in about 25% cases. The term “birdshot” is given because the pattern of lesions in the fundus resembles the shotgun scatter of a birdshot. A recent longitudinal cohort study has described the baseline clinical characteristics and has concluded that lesion pigmentation may be a marker of decreased visual function that is not reflected in central visual acuity.[73]

**POHS:** POHS is usually seen in endemic areas of **Histoplasma capsulatum and** rarely in other non-endemic areas.[1,74] It presents asymptomatically or with central scotoma. **H. capsulatum** has never been isolated from the choroid. CNVM is managed by argon laser photocoagulation for extrafoveal type, while krypton laser photocoagulation is beneficial for juxtafoveal type. Dabil *et al*.[75] reported a significant association between the HLA-DR15/HLA-DQ6 haplotype and development of CNVM in POHS

**Acute retinal pigment epithelitis (ARPE-Krill's disease)[76]:** It usually presents with unilateral blurred vision and metamorphopsia in young adults without significant prodromal flu-like illness. Round macular lesions, hallmark of the disease, are manifested by transient and subtle RPE alterations. Discrete clusters of small, hyperpigmented, dark gray spots at the RPE level surrounded by a yellowish white halo or area of macular depigmentation can affect vision. Gray dots and halo fade with resolution and become clinically undetectable. Vitritis is rare. VEP and ERG are normal, while EOG may be abnormal. FFA rules out CSR[51] and no treatment is needed.

### Treatment of non-infective posterior uveitic conditions

Systemic steroids are the mainstay of therapy in non-infective posterior uveitis. In vision-threatening lesions, such as those with optic nerve head or macular/foveal involvement, pulse therapy of intravenous methyl prednisolone (IVMP)[77] 1g daily for three consecutive days should be administered on an emergency basis only under care of an internist, preferably in place with an intensive care unit backup/set-up. This is followed by oral prednisolone 1–1.5 mg/kg/day, preferably single morning dose, in a tapering dosage schedule.

Once the diagnosis of non-infectious uveitis has been confirmed over time and infectious causes have been completely ruled out, intravitreal injections of steroid, such as triamcinolone or dexamethasone, are very useful adjuncts in controlling flare-ups.

In patients with repeated recurrences, intolerance to systemic steroids or recalcitrant to therapy, immunosuppressives can be added with/without systemic steroids.

Immunosuppressive options include antimetabolites such as azathioprine, methotrexate, and mycophenolate mofetil, T-cell suppressors such as cyclosporine and tacrolimus, and cytotoxic agents including cyclophosphamide and chlorambucil.

We usually prefer azathioprine,[63,78] considering the cost of therapy and as it has lesser side effects. It is given in a dose of 1–2 mg/kg/day in three divided doses and tapered monthly. A thorough knowledge of the usefulness and the side effects associated with the various immunosuppressives is a must before initiation of immunosuppressives.

The patient has to be explained the risks and benefits associated with the use of immunosuppressives and the need to monitor the side effects with the respective blood tests regularly.

The details regarding the usage of immunosuppressives and other details are described in other appropriate sections. Biologicals such as infliximab for the treatment of refractory non-infective posterior uveitis and severe SFU[69] and daclizumab and tacrolimus for the treatment of BCR[79–81] have been used with favorable response.

Local drug delivery such as intravitreal triamcinolone[82,83] for refractory CME is also effective. Inflammatory CNVM has been satisfactorily treated with intravitreal triamcinolone, bevacizumab[84–86] anecortave acetate in serpiginous choroiditis-associated CNVM,[87] and sirolimus for MFC-associated CNVM.[88] Fluocinolone acetonide, a local drug delivery implant, has been found to be useful in a large multicentre trial.[89–91]

Role of interferon-alpha[92] in severe, sight-threatening refractory uveitis is being considered, although adverse events like IFN-alpha-associated retinopathy may limit its use.

**Masquerade syndromes and other diseases[93,94]:** Masquerade syndromes are non-uveitic conditions that mimic and present like uveitis. It is important to differentiate neoplastic diseases from various other posterior uveitic entities.

Tumors such as lymphoma and other secondary malignancies can mimic viral retinitis. Lymphoma has to be ruled in non-resolving or atypical presentations of viral retinitis or intermediate uveitis. They are especially seen in patients with HIV infection. In children, one has to be aware of the possibility of leukemias and retinoblastoma, while in adults malignant melanoma and metastases are important differential diagnoses. Knowledge of non-uveitic entities, which can mimic posterior uveitis, is essential before initiating therapy.

A choroidal melanoma or metastases can be mistaken for a choroidal abscess. USG-B scan is very valuable to differentiate these entities. Choroidal melanoma shows a high to moderate surface reflectivity and variable low to moderate internal reflectivity on ultrasound. Other typical features include acoustic hollowing and choroidal excavation. It is generally associated with a shallow retinal detachment, although this may also be seen overlying a subretinal abscess.

Choroidal metastases appear as mass with high surface reflectivity and uniform high to moderate internal reflectivity. Acoustic hallowing and choroidal excavation are not present. They are usually associated with a large retinal detachment.

Vitreous and retinal biopsy is very useful in diagnostic dilemma.

**AIDS-related posterior uveitis[45]:** Infections such as toxoplasmosis, syphilis, and tuberculosis can have atypical ocular manifestations in AIDS. There are also some unique entities that give a clue to the underlying systemic disease. HIV retinopathy, rare tumors such as lymphomas, and infections such as cryptococcal and pneumocystis choroiditis are a few of them.

## Conclusions

Posterior uveitic entities have very characteristic clinical features and diagnosis is mainly clinical. It is essential to differentiate infective and non-infective conditions as their management is diametrically opposite. Infective posterior uveitic needs to be managed with specific anti-infective therapy and steroids. Empirical use of systemic steroids or immunosuppressives in all cases of posterior uveitis should be absolutely avoided. Judicial use of ancillary tests like FFA, ICG, USG, OCT, and electrophysiological tests as complimentary to clinical examination helps establish the right diagnosis. Intraocular fluid testing for PCR and antibodies in infective conditions is useful in diagnostic dilemma.[95–97] It is essential to follow-up all patients with posterior uveitis even after the resolution of lesions for complications related to the disease, such as CNVM, hemorrhage, or breaks. Macular or optic disc involvement can cause irreversible visual impairment and hence early diagnosis and appropriate management is important to save vision.
